# Supplementary material for: The Centriolar Adjunct–Appearance and Disassembly in Spermiogenesis and the Potential Impact on Fertility
Source: Cells. 2019 Feb 19;8(2):180. doi: 10.3390/cells8020180 (PMC6406449; doi:10.3390/cells8020180)
Supplement: Supplementary file 1 [file cells-08-00180-s001.pdf]

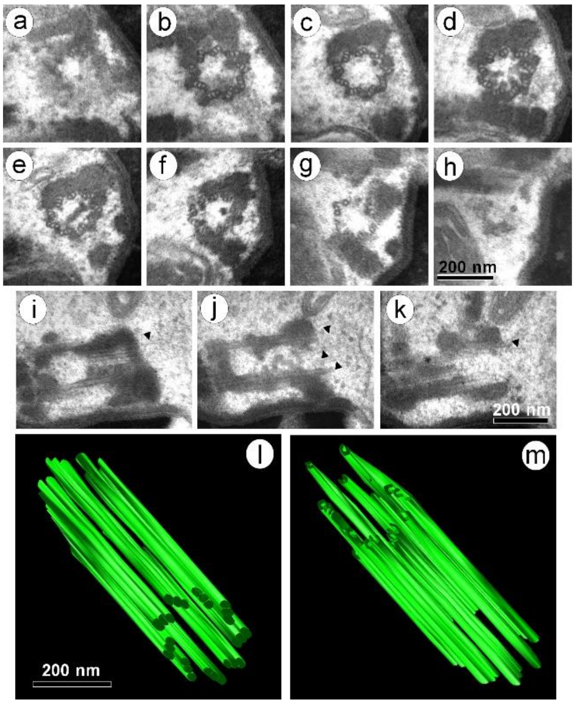

Figure S1. TEM images 8 cross (a-h) and 3 longitudinal (i-k) sections of PC+CA complexes in spermatozoa of fertile donor; l and m 3D reconstruction of PC+CA complex of fertile donor (two different angle of view). Scale bar 200 nm.

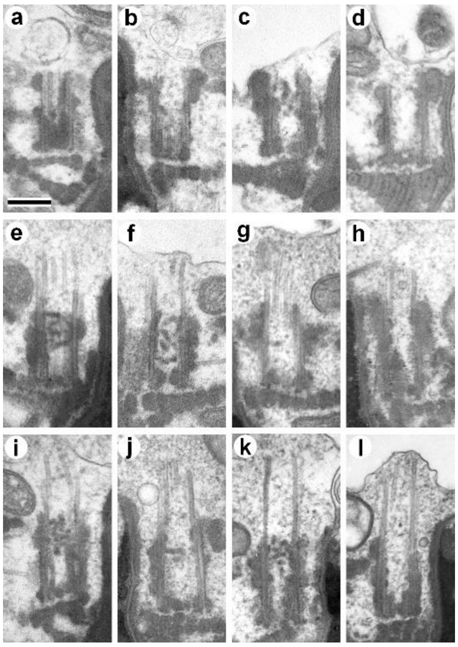

Figure S2. TEM images of PC+CA complexes. (a - c) Class I spermatozoa from D1. (d) Class I spermatozoid from P2. (e, f) Class II spermatozoa from D1. (g, h) Class II spermatozoa from P2. (i) Class III spermatozoid from D1. (j - l) Class III spermatozoa from P2. Scale bar 200 nm.

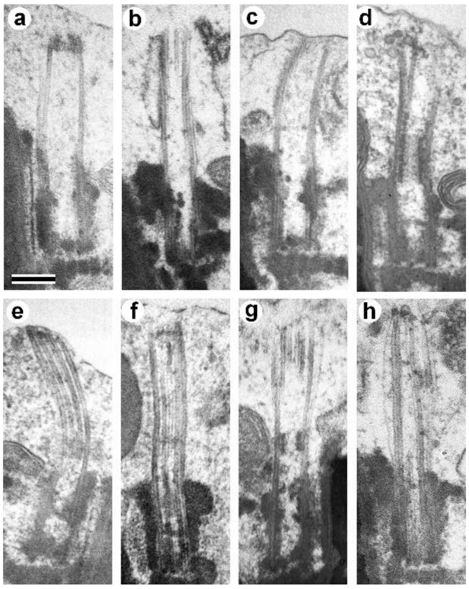

Figure S3. TEM images of PC+CA complexes. (a - c) Class IV spermatozoa from P1. (d) Class IV spermatozoa from P2. (e - h) Class V spermatozoa from P1. Scale bar 200 nm.
